# Molecular Heterogeneity Between Paired Primary and Metastatic Lesions from Clear Cell Renal Cell Carcinoma

**DOI:** 10.1016/j.euros.2022.04.004

**Published:** 2022-05-02

**Authors:** Eduard Roussel, Lisa Kinget, Annelies Verbiest, Jessica Zucman-Rossi, Bram Boeckx, Steven Joniau, Diether Lambrechts, Maarten Albersen, Benoit Beuselinck

**Affiliations:** a Department of Urology, University Hospitals Leuven, Leuven, Belgium; b Department of General Medical Oncology, University Hospitals Leuven, Leuven Cancer Institute, Leuven, Belgium; c Inserm UMR-1162, Génomique fonctionelle des tumeurs solides, Institut Universitaire Hématologie, Paris, France; d Laboratory of Translational Genetics, Department of Human Genetics, KU Leuven, Leuven, Belgium; e VIB Center for Cancer Biology, Leuven, Belgium

**Keywords:** Renal cell carcinoma, Clear cell, Heterogeneity, Molecular subtypes, Metastases

## Abstract

Highly effective systemic treatments have globally improved outcomes in metastatic clear-cell renal cell carcinoma (m-ccRCC). However, despite many efforts, reliable biomarkers predicting individual responses are currently lacking. Moreover, mixed responses are commonly observed. We hypothesized that molecular heterogeneity between primary tumors and their metastases could flaw biomarker research based on features of the primary tumor and explain mixed responses. Therefore, we studied the heterogeneity of the ccrcc1–4 molecular subtypes across patient-matched primary and metastatic lesions over time in 62 patients with m-ccRCC who underwent both nephrectomy and metastasectomy. These subtypes characterize underlying disease biology and are associated with outcomes in both the primary and metastatic settings. We observed a concordance rate of 58% (95% confidence interval 45–71%). This concordance was not affected by the interval between nephrectomy and resection of the metastatic lesion. Across discordant pairs, the metastatic lesions mostly exhibited a less favorable molecular subtype. Moreover, primary tumors with the favorable ccrcc2 molecular subtype were characterized by favorable prognosis and a long interval between nephrectomy and metastasectomy. Conversely, tumors with the unfavorable ccrcc4 molecular subtype relapsed quickly and had poor prognosis. Thus, the considerable molecular heterogeneity between patient-matched m-ccRCC primary and metastatic lesions provides an explanation for mixed responses to systemic therapy and could impact the development of biomarker studies in which the primary tumor is often considered a surrogate for metastatic disease.

**Patient summary:**

We studied primary tumors and metastases from patients with kidney cancer and found considerable heterogeneity in their molecular features. This heterogeneity explains mixed responses to systemic therapy and is important to take into account in future biomarker studies for this disease.

In recent years, novel and highly effective systemic treatments for metastatic clear-cell renal cell carcinoma (m-ccRCC) have revolutionized the treatment landscape and globally improved outcomes for patients [1]. However, despite many efforts, reliable biomarkers predicting the response in individual patients are currently lacking. Moreover, mixed responses are commonly observed. A better understanding of molecular heterogeneity between primary tumors and their metastases is crucial to predict these response patterns and ultimately guide biomarker development with respect to personalized medicine.

Molecular subtypes have emerged as a way to characterize underlying disease biology and potentially guide therapeutic decision-making. We previously described four molecular subtypes of ccRCC that are associated with outcomes in both the localized and metastatic settings for patients receiving systemic therapy and patients undergoing surgical metastasectomy [2], [3], [4], [5]. In brief, we found that the ccrcc2 and ccrcc3 subtypes have favorable prognosis, with the ccrcc2 subtype characterized by heightened angiogenesis-related gene expression signatures and better responses to angiogenesis inhibitors. Tumors of the ccrcc3 subtype exhibit an expression profile similar to that of normal kidney tissue. The ccrcc1 and ccrcc4 subtypes have intermediate and poor prognosis, respectively. While they both show relative upregulation of Myc targets, the ccrcc4 subtype is characterized by high expression of immune-related gene expression signatures and is enriched in sarcomatoid features, a feature associated with better response to immune checkpoint inhibition [2], [3], [4].

Brannon et al. [6] described the ClearCode34 ccA and ccB molecular subtypes, which are associated with favorable and unfavorable prognosis, respectively. Serie et al. [7] studied the ClearCode34 ccA and ccB molecular subtypes in 81 patient-matched primary and metastatic lesions and reported that these subtypes differed 43% of the time between the primary tumor and the metastasis. Thus, when comparing two molecular subtypes, the chance of finding a concordant molecular subtype was close to the result for flipping a coin, suggesting that the primary tumor might not be a good surrogate for the metastatic lesions at all. Therefore, we aimed to provide more granular data on heterogeneity between primary tumors and matched metastatic lesions by studying the four ccrcc molecular subtypes of primary tumors and patient-matched metastases over time.

We collected archived formalin-fixed, paraffin-embedded (FFPE) tissue specimens from 62 patients with m-ccRCC who underwent nephrectomy at our institution between 1998 and 2018 and who had tissue available from both the primary tumor and at least one metastasis. We performed whole-transcriptome RNA sequencing to determine the ccrcc molecular subtypes for the 62 primary tumors and 121 metastases as previously described [3], [4]. Of note, all metastasis tissue was obtained via surgical metastasectomy, which entails a general selection bias towards patients with more favorable characteristics [8].

The baseline characteristics of our cohort are shown in Table 1. Of the primary tumors, 13 (21.0%) were ccrcc1, 40 (64.5%) were ccrcc2, one (1.6%) was ccrcc3, and eight (12.9%) were ccrcc4. Fig. 1 shows a swimmer plot depicting the resected metastases over time and their ccrcc molecular subtype. Clearly, patients with a primary tumor of the favorable ccrcc2 subtype are characterized by a clinical disease course with long overall survival (median 74 mo) and a long interval between resection of the primary tumor and of the metastatic lesion(s) (median 19 mo). Conversely, patients with a ccrcc4 primary tumor had poor overall survival (median 20 mo) and a short time between resection of the primary and the metastasis (median 0 mo).

**Table 1:** Baseline characteristics

Parameter	Result
Patients (*n*)	62
Median age at nephrectomy, yr (interquartile range)	62 (53–67)
Male, *n* (%)	44 (71.0)
Synchronous metastases at nephrectomy, *n* (%)	27 (43.6)
Median number of metastases resected, *n* (interquartile range)	1 (1–2)
Molecular subtype of the primary tumor, *n* (%)	
ccrcc1	13 (21.0)
ccrcc2	40 (64.5)
ccrcc3	1 (1.6)
ccrcc4	8 (12.9)
Metastases (*n*)	121
Molecular subtype of metastases, *n* (%)	
ccrcc1	12 (9.9)
ccrcc2	66 (54.6)
ccrcc3	5 (4.1)
ccrcc4	38 (31.4)
Location of resected metastases, *n* (%)	
Lung	32 (26.5)
Nonregional lymph nodes	25 (20.7)
Contralateral kidney	10 (8.3)
Contralateral adrenal gland	10 (8.3)
Bone	10 (8.3)
Pancreas	6 (5.0)
Brain	4 (3.3)
Thyroid	4 (3.3)
Gingiva	3 (2.5)
Ipsilateral retroperitoneum	3 (2.5)
Skin	3 (2.5)
Intestine	2 (1.7)
Liver	2 (1.7)
Pleura	2 (1.7)
Gall bladder	1 (0.8)
Ovary	1 (0.8)
Ureter	1 (0.8)
Peritoneal	1 (0.8)
Epidural	1 (0.8)

**Fig. 1:**
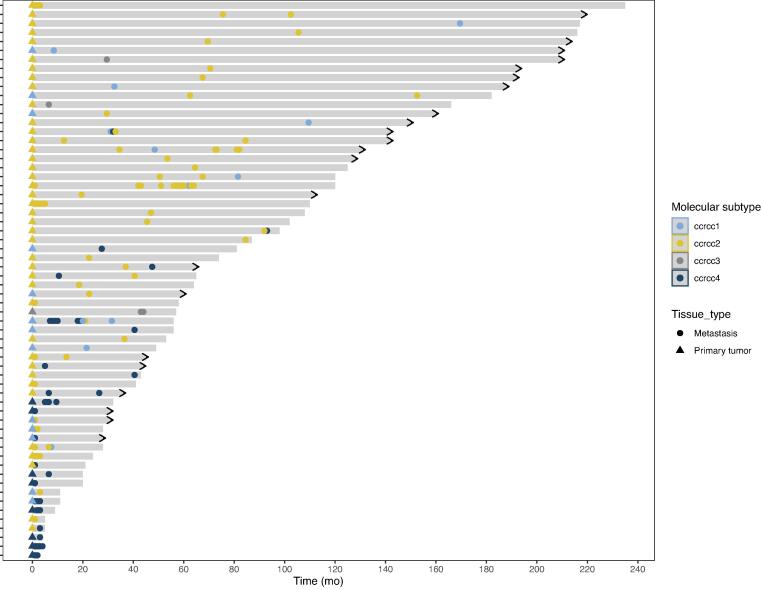
Swimmer plot depicting results for 62 patients with formalin-fixed, paraffin-embedded tissue available from both the primary tumor and at least one metastatic lesion. Each line represents a single patient. The length of the grey bars represent the overall survival of each patient following nephrectomy, with an arrowhead at the end indicating whether the patient is still alive. The symbols represent the timing and ccrcc molecular subtype of each surgical metastasectomy specimen.

We calculated the ccrcc molecular subtype concordance between the primary tumor and the first resected metastasis. If multiple metastases resected at the same time point had discrepant molecular subtypes, the observation was considered discordant. The 95% confidence intervals (CIs) were calculated using the Clopper-Pearson exact method.

Out of the 62 pairs, 36 (58%) were concordant (95% CI 45–71%). Out of the 26 discordant pairs, 18 (69.2%) switched to a more unfavorable subtype in at least one of the resected metastases, while six (23.1%) switched to a more favorable subtype and two (7.7%) switched between the ccrcc2 and ccrcc3 subtypes, which both have favorable prognosis. The fact that discordant subtypes more often switch to a more aggressive subtype is in line with the Serie study which reported a switch from the favorable ccA to the unfavorable ccB subtype in 80% of discordant cases.

However, concordance analysis does not consider the effect of time. To assess whether the concordance rate depends on the time between resection of the primary tumor and the metastasis, we performed logistic regression with molecular subtype concordance between the primary tumor and metastasis as the dependent variable and time as the independent variable. This showed no significant impact of the time to the first metastasectomy on the concordance (odds ratio 1.0, 95%CI 0.98–1.01; *p* = 0.79).

We did not assess molecular heterogeneity within the same primary tumor in our study, which poses an additional challenge for routine use of tissue-based biomarkers. This issue might be addressed in the future with the use of imaging-based biomarkers, providing whole-tumor virtual biopsies [9]. Moreover, it has been shown that tumors with higher intratumoral heterogeneity often exhibit a disease course that is characterized by attenuated progression and gradual acquisition of metastatic competence [10]. Conversely, primary tumors with low intratumoral heterogeneity and a monoclonal structure often exhibit rapid disease progression to multiple disease sites.

Thus, while our study is limited by the long time period for patient inclusion, the fact that all patients included underwent both nephrectomy and metastasectomy provides more granular data on the considerable heterogeneity between primary tumors and matched metastases from m-ccRCC. This heterogeneity might provide an explanation for mixed responses to systemic therapy. Moreover, this is an important consideration for the development of biomarker studies in which the primary tumor is considered a surrogate for metastatic lesions and our findings underscore the value of tissue sampling of metastatic sites if tissue-based biomarkers might direct therapeutic decision-making in the future.

  ***Author contributions***: Eduard Roussel had full access to all the data in the study and takes responsibility for the integrity of the data and the accuracy of the data analysis.

Study concept and design: Roussel, Beuselinck.

*Acquisition of data*: Roussel, Verbiest, Kinget, Beuselinck.

*Analysis and interpretation of data*: Roussel.

*Drafting of the manuscript*: Roussel.

*Critical revision of the manuscript for important intellectual content*: Roussel, Kinget, Verbiest, Zucman-Rossi, Boeckx, Joniau, Lambrechts, Albersen, Beuselinck.

*Statistical analysis*: Roussel.

*Obtaining funding*: Beuselinck.

*Administrative, technical, or material support*: None.

*Supervision*: Albersen, Beuselinck.

*Other*: None.

  ***Financial disclosures:*** Eduard Roussel certifies that all conflicts of interest, including specific financial interests and relationships and affiliations relevant to the subject matter or materials discussed in the manuscript (eg, employment/affiliation, grants or funding, consultancies, honoraria, stock ownership or options, expert testimony, royalties, or patents filed, received, or pending), are the following: None.

  ***Funding/Support and role of the sponsor*:** This research was funded by Bristol-Myers Squibb. The sponsor played a role in the design and conduct of the study and approval of the manuscript.
